# Rapid Transcriptional Reprogramming Triggered by Alteration of the Carbon/Nitrogen Balance Has an Impact on Energy Metabolism in *Nostoc* sp. PCC 7120

**DOI:** 10.3390/life10110297

**Published:** 2020-11-20

**Authors:** Peter J. Gollan, Dorota Muth-Pawlak, Eva-Mari Aro

**Affiliations:** Department of Biochemistry, Molecular Plant Biology, University of Turku, Tykistökatu 6A, 20520 Turku, Finland; dokrmu@utu.fi (D.M.-P.); evaaro@utu.fi (E.-M.A.)

**Keywords:** cyanobacteria, *Nostoc* sp. PCC 7120, transcriptomics, photosynthesis, carbon/nitrogen

## Abstract

*Nostoc* (*Anabaena*) sp. PCC 7120 is a filamentous cyanobacterial species that fixes N_2_ to nitrogenous compounds using specialised heterocyst cells. Changes in the intracellular ratio of carbon to nitrogen (C/N balance) is known to trigger major transcriptional reprogramming of the cell, including initiating the differentiation of vegetative cells to heterocysts. Substantial transcriptional analysis has been performed on *Nostoc* sp. PCC 7120 during N stepdown (low to high C/N), but not during C stepdown (high to low C/N). In the current study, we shifted the metabolic balance of *Nostoc* sp. PCC 7120 cultures grown at 3% CO_2_ by introducing them to atmospheric conditions containing 0.04% CO_2_ for 1 h, after which the changes in gene expression were measured using RNAseq transcriptomics. This analysis revealed strong upregulation of carbon uptake, while nitrogen uptake and metabolism and early stages of heterocyst development were downregulated in response to the shift to low CO_2_. Furthermore, gene expression changes revealed a decrease in photosynthetic electron transport and increased photoprotection and reactive oxygen metabolism, as well a decrease in iron uptake and metabolism. Differential gene expression was largely attributed to change in the abundances of the metabolites 2-phosphoglycolate and 2-oxoglutarate, which signal a rapid shift from fluent photoassimilation to glycolytic metabolism of carbon after transition to low CO_2_. This work shows that the C/N balance in *Nostoc* sp. PCC 7120 rapidly adjusts the metabolic strategy through transcriptional reprogramming, enabling survival in the fluctuating environment.

## 1. Introduction

Cyanobacteria use light energy to fix inorganic carbon (C_i_) and nitrogen (N), harvested from their aquatic environment, into the metabolic components required for growth and propagation. Environmental sources of C_i_ include dissolved CO_2_ and bicarbonate (HCO_3_^−^), while N can be supplied by nitrate (NO_3_^−^), nitrite (NO_2_^−^), ammonium (NH_4_^+^), urea or N_2_ (in diazotrophic cyanobacteria; reviewed in [1]). The tight coupling of the concentrations of C_i_ and N taken up from the environment prevents metabolic imbalance within the cell, which allows cyanobacteria to thrive amidst varying nutritional conditions. This is achieved in large part by transcriptional modifications that are triggered by fluctuations in the cellular homeostasis of organic carbon (C) and N, which are represented by changes in the relative abundances of key metabolite signals (reviewed in [2,3,4]). One such metabolite is 2-oxoglutarate (2OG), also known as α-ketoglutarate (αKG), which is a product of the tricarboxylic acid (TCA) cycle. The metabolite 2OG provides the inorganic carbohydrate skeleton for glutamate synthesis that occurs by the incorporation of NH_4_^+^ in the glutamine synthetase/glutamine-oxoglutarate aminotransferase (GS/GOGAT) cycle. Cellular 2OG levels therefore represent the abundances of both C and N (reviewed in [5,6]), making 2OG a central signalling metabolite that triggers transcriptional adjustments to restore C/N balance [7,8,9]. 2-phosphoglycolate (2PG) is another metabolite that controls transcriptional reprogramming in response to C/N balance [10], and 2PG is formed when Rubisco catalyses the oxygenation of RuBP (photorespiration), instead of the favoured carboxylation reaction between RuBP and CO_2_ (reviewed in [4,11]). An increase in 2PG concentration therefore represents CO_2_ deficiency, triggering transcriptional reprogramming designed to upregulate C_i_ import into the cell [12,13,14,15].

*Nostoc* (*Anabaena*) sp. PCC 7120 is a filamentous, diazotrophic cyanobacterium wherein C/N balance controls the formation of heterocyst cells specialised for fixing N_2_ into NH_4_^+^, while photosynthetic CO_2_ fixation is restricted to vegetative cells (reviewed in [16,17]). In *Nostoc* sp. PCC 7120 and other heterocystous cyanobacteria, differentiation in cell structure and function is triggered by changes in C/N homeostasis and enacted by massive transcriptional reprogramming [1]. Emphasis on the impact of N concentration on heterocyst differentiation has revealed the central roles of 2OG and several regulatory proteins in instigating transcriptional and physiological responses to N deficit [6]. However, the transcriptional response of heterocystous cyanobacteria to C_i_ availability has drawn only little attention [18], in sharp contrast to that in non-diazotrophic species [12,13,15,19,20,21,22]. In the current study of the global transcriptome of *Nostoc* sp. PCC 7120, we found that a shift from 3% CO_2_ to 0.04% CO_2_ for 1 h can be largely attributed to changes in the levels of the metabolites 2PG and 2OG, which has a strong effect on genes involved in the import and metabolism of C_i_ and N. This study also identified that genes encoding factors involved in photosynthetic electron transport, glycolysis and iron homeostasis are regulated by C/N homeostasis, which is suggested to trigger a transition from efficient photoautotrophic growth and energy storage to photoinhibition and glycolysis.

## 2. Materials and Methods

### 2.1. Growth and CO_2_ Stepdown

*Nostoc* sp. PCC 7120 cultures were grown in BG11 medium [23] buffered with 10 mM TES-KOH (pH 8.0) at 30 °C under constant illumination of 50 μmol photons m^−2^ s^−1^ with 120 rpm agitation, in air enriched with 3% (*v*/*v*) CO_2_. During the exponential growth phase (OD_750_ = 1.0), 2 mL samples were taken from three individual replicate cultures and frozen for RNA isolation. For CO_2_ stepdown, the cultures were pelleted and the pellets washed once with fresh BG11, before resuspension in fresh BG11 and growth in air containing 0.04% (*v*/*v*) CO_2_. After 1 h, 2 mL samples were collected from three replicates and frozen for RNA isolation.

### 2.2. RNA Isolation and Transcriptomics

Total RNA was isolated as described in [24]. Total RNA samples were submitted to the Beijing Genomics Institute (China) for library construction and RNA sequencing using Illumina HiSeq2500. RNA reads were aligned using Strand NGS 2.7 software (Avadis) using the *Nostoc* sp. PCC 7120 reference genome and annotations downloaded from Ensembl (EBI). Aligned reads were normalised and quantified using the DESeq package (R). Significantly differentially expressed genes were identified using a 2-way ANOVA. *p*-Values were adjusted for false discovery rate (FDR) using the Benjamini–Hochberg procedure.

## 3. Results

The transcriptome of *Nostoc* sp. PCC 7120 grown in BG11 under 3% CO_2_-enriched air was compared with that of the same strain shifted from 3% CO_2_ to 0.04% CO_2_ for 1 h, revealing 230 genes to be upregulated >2-fold and 211 genes to be downregulated >2-fold, in the low CO_2_ condition. The RNAseq data are available at the NCBI Sequence Read Archive (submission SUB8244772). As expected, given the short period under a new metabolic condition, no differences in the growth rates, lengths of filaments or frequencies of heterocysts (approximately 4% of all cells under 3% CO_2_) were observed between the cultures exposed to 0.04% CO_2_ conditions, compared to those grown at 3% CO_2_. Therefore, statistical tests of these parameters in the different cultures were not performed.

### 3.1. Uptake and Metabolism of Carbon and Nitrogen Are Inversely Responsive to Low CO_2_ Conditions

The operons encoding three plasma membrane-localised HCO_3_^−^ uptake systems were among the most strongly upregulated entities in *Nostoc* sp. PCC 7120 following CO_2_ stepdown (Table 1). In cyanobacteria, the Cmp (BCT1) system is powered by ATP hydrolysis [25], while the SbtA and BicA systems depend on Na^+^ ions for HCO_3_^−^ symport [21,26]. The upregulation of the operon encoding the Mrp Na^+^:H^+^ antiporter upon CO_2_ stepdown may also be linked to HCO_3_^−^ uptake through the extrusion of Na^+^ to support SbtA and BicA activity [27,28]. HCO_3_^−^ uptake in cyanobacteria forms a major part of the carbon concentration mechanism (CCM), which also involves the concentration of cellular CO_2_ into HCO_3_^−^ by a customised NAD(P)H dehydrogenase (NDH-1) complex [29]. Genes *ndhF3*, *ndhD3* and *cupA*, which encode subunits that specialise the NDH1–MS complex for inducible CO_2_ uptake, were upregulated after CO_2_ stepdown (Table 1), as were several other genes encoding the core NDH–1M complex (see below). Notably, a putative *cupS* orthologue (*alr1320*), which is encoded separately from the *ndhF3*/*ndhD3*/*cupA* cluster in the *Nostoc* sp. PCC 7120 genome [30], was not differentially expressed (DE) in the current work. Genes encoding Rubisco and some carboxysome subunits were mildly upregulated in low CO_2_ (1.3 to 1.7 fold change (FC); not shown), while other CCM components were not DE, suggesting that these components were already in sufficient abundance before CO_2_ stepdown, whereas C_i_ uptake from the environment, especially HCO_3_^−^ uptake, was apparently a primary concern for survival after 1 h under low CO_2_.

The decrease in CO_2_ was found to cause downregulation of the *nir* operon, which encodes subunits of an ATP-dependent nitrate (NO_3_^−^) transporter, as well as NO_3_^−^ and nitrite (NO_2_^−^) reductases [31,32]. The *nir* operon is responsible for NO_3_^−^ uptake and reduction to ammonia (NH_3_), and is broadly conserved across cyanobacteria (reviewed in [5]). Unlike the *nir* operon, the majority of *nif* genes that encode subunits for the assembly and function of nitrogenase, which reduces atmospheric N_2_ to NH_3_, were not DE in the current work. Exceptions were *nifB* and *nifH2*, which were downregulated (Table 1). A gene that encodes a protein similar to the C-terminus of Mo-like nitrogenase (*alr1713*) was strongly downregulated, along with its neighbour (*asr1714*); however, the function of the encoded proteins is not known. Since heterocyst development is upregulated by a high C/N ratio (reviewed in [33]), it was not surprising to see many genes involved in the structural development of heterocysts repressed by the shift to low CO_2_. In particular, the *hpd*, *hgl* and *dev* gene clusters that encode many components for the synthesis and export of glycolipids, which form the oxygen-impermeable heterocyst envelope (reviewed in [34,35]), and were downregulated in the current data. Notably, the *hep* genes encoding heterocyst outer layer polysaccharides were only moderately downregulated by the shift to low CO_2_ (average FC −1.5, data not shown).

### 3.2. Expression of Genes Encoding Photosynthetic and Respiratory Components Responds to Low CO_2_ Conditions

Expression of genes encoding photosystem II (PSII) core proteins D1 and D2 was upregulated after the shift to low CO_2_ (Table 2), indicating an increase in the damage and turnover of PSII reaction centres [36,37]. In keeping with this, the genes encoding the two FtsH proteases involved in the degradation and turnover of damaged D1 protein were also upregulated [38,39]. We also found strongly induced expression of the *flv2–flv4* operon, as well as several genes encoding orange carotenoid proteins (OCPs) and early light-inducible proteins (ELIPs), all of which are associated with PSII photoprotection [40,41,42,43,44]. These expression data suggest that the shift to low CO_2_ led to the over-reduction, damage and repair of PSII. Given this evidence for PSII over-reduction, it was surprising to find the gene encoding the “plastid” terminal oxidase (PTOX) as one of the most strongly downregulated in the current study (Table 2), being highly expressed under 3% CO_2_ and strongly repressed in 0.04% CO_2_. PTOX is part of a water–water cycle that moves electrons from reduced plastoquinone (PQ) to O_2_, and is thought to be an electron valve for balancing the photosynthetic redox state [45], which would presumably be important under low CO_2_ (discussed below).

Virtually all genes encoding subunits of photosystem I (PSI) were substantially downregulated in the current study, which is in contrast to their increased expression in *Synechocystis* sp. PCC 6803 and unchanged expression *Synechococcus elongatus* PCC 7942 in low CO_2_ [13,20]. PSI downregulation in *Nostoc* sp. PCC 7120 points towards a decrease in PSI electron transport that may be related to the diminution of the terminal electron acceptor CO_2_. These conditions would also be expected to upregulate O_2_ reduction and the subsequent formation of toxic superoxide radicals (O_2_^•−^); indeed, genes encoding a superoxide dismutase (SodB; *alr2938*) and peroxiredoxin (*all2375*), involved in reactive oxygen species (ROS) scavenging, were upregulated 3.2-fold and 2.3-fold, respectively (not shown). The expression of *isiB* (*alr2405*), which encodes a flavodoxin (Fld) that accepts electrons from PSI via a flavin mononucleotide cofactor [46], was also upregulated after low CO_2_ treatment (Table 2), suggesting a shortage of oxidised ferredoxin (Fd) acceptors [47]. The upregulated expression of genes encoding F_0_-F_1_ ATP synthase points to an increased demand for energy in low CO_2_, required for HCO_3_^−^ import and CO_2_ hydration (reviewed in [2]). Notably, the gene *alr1004* encoding an enzyme that converts glyoxylate to glycine for the detoxification of 2PG [18] was found to be downregulated after CO_2_ stepdown, while other enzymes in the glycolate metabolism pathway were not DE.

The downregulation of PSI abundance in response to low CO_2_ can partially clarify the apparent PSII over-reduction discussed above. Lower PSI levels may also be linked to a decrease in the number of heterocysts, which contain a higher PSI:PSII ratio than in vegetative cells [16]. Although such a decrease in heterocysts was not observed after 1 h in low CO_2_, suppressed heterocyst development was evident in the downregulation of *hgl* and *dev* clusters (Table 1), and this can also explain the suppression of genes encoding cytochrome c6, Flv1B and Flv3B (Table 2) that operate predominately [48] or exclusively [49] in heterocysts. The gene encoding the small subunit of the heterocyst-specific uptake hydrogenase (HupS) was also downregulated here, reflecting the downregulation of the heterocystous nitrogenase activity under low CO_2_.

We observed downregulation of the *pec* cluster that encodes the phycoerythrocyanin (PEC) parts of the light-harvesting phycobilisome (PBS) complex [50,51], while genes encoding the other components of the PBS were not DE, indicating that the light-harvesting cross-section of PBS in *Nostoc* sp. PCC 7120 is altered in response to low CO_2_. An operon encoding a subunit of the light-independent protochlorophyllide reductase (DPOR) was also downregulated after 1 h in low CO_2_, while the expression of *chlG* and *hemH* genes, involved in later stages of chlorophyll and haem synthesis, respectively, were upregulated (Table 2).

Most genes encoding subunits of the NDH-1 complex were upregulated under low CO_2_ (Table 2), probably to fulfil their role in CO_2_ uptake as part of the NDH–1MS complex described above. In contrast, *ndbA* encoding NDH-2 was downregulated in the current study, suggesting a decrease in NDH-2-mediated respiration after the shift to low CO_2_. In addition, the gene encoding phosphoenolpyruvate (PEP) synthase, which converts pyruvate to PEP that is consumed in the TCA cycle, was strongly downregulated (Table 2). Many genes encoding subunits of the bidirectional hydrogenase (Hox) were among the most strongly downregulated in response to low CO_2_ conditions (Table 2), being both highly expressed in 3% CO_2_ and strongly repressed in low CO_2_. Hox reversibly catalyses the reduction of H^+^ to form H_2_, powered by reduced Fd/Fld with electrons derived from either PSI or pyruvate, the latter route by way of pyruvate ferredoxin/flavodoxin oxidoreductase (PFOR), which converts pyruvate to acetyl-CoA (reviewed in [52]). The *nifJ* gene encoding PFOR followed a similar expression profile to Hox subunits, being another of the most strongly downregulated genes in the current study. The physiological role of the Hox enzyme is not known, but has been described as an electron valve that can maintain redox balance and store reducing power as H_2_ during excess photosynthesis or fermentation [52,53,54,55]. The expression profile of Hox and PFOR genes suggests that pyruvate-powered hydrogen production is active under 3% CO_2_ and inactivated by the shift to low CO_2_.

### 3.3. Expression of Transcription Regulators Responds to Changes in CO_2_ Conditions

The current study revealed substantial changes in the expression of several genes encoding transcription regulators, providing evidence of an ongoing cascade of transcriptional reprogramming after 1 h under low CO_2_ (Table 3). Upregulated expression of the LysR-type regulator (LTTR) *cmpR* corresponds to the strong upregulation of its target, the *cmp* cluster encoding the BCT1 HCO_3_^−^ transporter (Table 1), as previously shown in *Synechocystis* sp. PCC 6803, *Synechococcus* sp. PCC 7942 [56] and *Nostoc* sp. PCC 7120 [57]. Two sigB-type group 2 sigma factors, which have roles in the transcriptional response to low CO_2_ and C/N balance [58,59,60], were upregulated after the shift to low CO_2_ (Table 3). SigB has also been implicated in response to environmental stress and resistance to photoinhibition in *Synechocystis* sp. PCC 6803 [61,62], which is in line with the upregulation in this study of *groES* and *groEL* genes (4.3 to 5.5 FC; not shown) and photoprotective factors such as OCPs and the *flv2–flv4* operon (Table 2). Two homologous two-component response regulator clusters, which each comprise a histidine kinase and a DNA-binding regulator, were found to be upregulated by low CO_2_. Of the two, the chromosomal *hik31* (*C-hik31*) operon was more highly upregulated, and has been found to be involved in the responses to oxygen concentration, light and metabolism [63,64]. A TetR-family transcription regulator with unknown function was also upregulated by low CO_2_ (Table 3).

Another LTTR gene that was highly expressed under high CO_2_ and was strongly downregulated after low CO_2_ transition (Table 3) shared substantial sequence homology with the *ndhR* transcription repressor (also called *ccmR*) of *Synechocystis* sp. PCC 6803 [12,27]. In other cyanobacteria, NdhR represses the expression of CCM genes, including the *sbt* and *bicA* HCO_3_^−^ importers, the *mrp* cluster and the *ndhF3*/*ndhD3*/*cupA* cluster [12,15,20,27,65]. The rapid downregulation of a putative *ndhR* orthologue in *Nostoc* sp. PCC 7120 may reveal the mechanism behind the strong upregulation of CCM genes after CO_2_ stepdown in the current study (Table 1). Downregulation of the transcription enhancer *ntcB*, which increases N metabolism through upregulation of the *nir* operon [66], also correlates with downregulation of other N-related genes in the current work (Table 1). Expression of *ntcB* is controlled by NtcA [1]. As *ntcA* was not DE in the current study, downregulation of *ntcB* and other NtcA regulons may be due to the low CO_2_-induced inactivation of NtcA (discussed below). Similarly, downregulated transcriptional regulator genes *patB* (also called *cnfR*), *devH* and *nrrA* can also be attributed to inhibited NtcA activity [67,68,69,70,71]. These genes are expressed in heterocysts, where PatB upregulates the expression of *nifB* [72], DevH regulates heterocyst glycolipids [48,68,73] and NrrA induces expression of both the heterocyst regulator *hetR* and *fraF* encoding a filament integrity protein [70,74]. Both *nifB* and the *hgl* cluster were downregulated in this study (Table 1), while *hetR* and *fraF* were not DE (not shown).

### 3.4. Low CO_2_ Conditions Influence the Expression of Metal Homeostasis Genes

A number of genes and gene clusters related to cellular homeostasis of iron (Fe) and other metals were found to be DE after CO_2_ stepdown (Table 4). A gene cluster encoding subunits of a periplasmic ferrous Fe (Fe(II)) transporter [75,76] was strongly downregulated in the current study (Table 4), indicating a lower uptake of Fe from the environment under low CO_2_. The *suf* cluster, encoding proteins involved in Fe mobilization and Fe–S cluster assembly, was also downregulated. The expression of both the Fe(II) transporter and the *suf* cluster is upregulated by Fe deprivation [77,78,79], suggesting a surplus of cellular Fe after low CO_2_ treatment. Several neighbouring clusters of genes encoding subunits of metal cation efflux systems such as copper, nickel, zinc, cadmium and cobalt, were upregulated after low CO_2_ treatment (Table 4). These genes have been implicated in heavy metal resistance [80,81], although the link to the current conditions is not clear. Taken together, low CO_2_ appears to induce an active decrease in cellular metal content, which may be a strategy to avoid oxidative stress during the reducing conditions induced by an insufficient availability of photosynthetic electron acceptors.

## 4. Discussion

### 4.1. Transcriptional Regulation in Response to CO_2_ Stepdown is Triggered by Metabolites

Induction of the most strongly upregulated genes, the HCO_3_^−^ transporters (Table 1), after CO_2_ stepdown, suggests a rapid transcriptional response that is highly sensitive to cellular C_i_ levels. In some unicellular cyanobacteria, repression of the CCM genes by NdhR (also called CcmR) can be modulated by both 2OG and 2PG [12,15,65,82]. Increased cellular 2PG concentration caused by increased photorespiration in low CO_2_ leads to increased abundance of the NdhR–2PG complex that is unable to bind DNA to repress expression [4]. Additionally, declining 2OG levels due to lower CO_2_ fixation and potentially lower TCA cycle activity decrease the abundance of the NdhR-2OG repressor complex, although it is unclear whether 2OG levels would actually decrease after only 1 h in low CO_2_ due to the mobilisation of stored glycogen into the TCA cycle [2,15,82,83]. NADP^+^, another co-repressor of NdhR [82], is also theoretically far less abundant after CO_2_ stepdown, due to decreased CO_2_ fixation and lower NADPH consumption despite constant light conditions. Although a putative NdhR in *Nostoc* sp. PCC 7120 has not been studied, it appears that the LTTR encoded by *all4986* represents such an orthologue, and that the strong downregulation of *all4986* after CO_2_ stepdown led to de-repression of the NdhR regulon, which includes the *ndhR* gene itself [84]. Previous transcriptomics studies have shown *ndhR* expression to be upregulated in *Synechocystis* sp. PCC 6803 after >3 h in low C_i_ conditions [12,13], which is in contrast to the strong downregulation of *all4986* seen here after 1 h (Table 3). This suggests that NdhR de-repression in response to CO_2_ stepdown may be transient, and/or that expression of the NdhR regulon is also controlled by other transcription factors [18]. In the current study, de-repression by the putative NdhR is proposed to have caused a rapid and strong increase in HCO_3_^−^ and CO_2_ uptake under C limitation, with the upregulated *cmp* operon (Table 1) inducing further increase in HCO_3_^−^ uptake. The *cmp* inducer CmpR is activated by 2PG or RuBP [82,85], both of which are in higher concentrations after CO_2_ stepdown due to decreased CO_2_ fixation. Furthermore, *cmpR* expression is also auto-upregulated (Table 3) [57]. In *Synechococcus* sp. PCC 7942 CmpR additionally upregulates the expression of PSII core subunits [86,87], found upregulated also in the current study alongside factors for PSII photoprotection and turnover, and downregulation of most PSI genes (Table 2). The overlap between cellular responses to either low CO_2_ or high light stress is well documented [12,20,86,88], highlighting insufficient electron sinks similarly created by both high light and low CO_2_, and resulting in the over-reduction of photosynthetic electron carriers [2]. Notably, several PSII photoprotection factors encoded by genes that were DE in the current study, including *psbAIII*, *flv4* and *sodB*, were shown to be regulated together with Rubisco and other CCM genes by another LTTR in *Nostoc* sp. PCC 7120 called PacR [18], suggesting the likely involvement of PacR in the transcriptional reprogramming seen here.

In addition to the LTTR transcription factors, the transcriptional response to low CO_2_ is also regulated by LexA and the cyAbrB paralogues [89,90,91,92]. The vast change in expression of the *hox* operon after CO_2_ stepdown (Table 2) may be related to the activity of LexA [93] and/or cyAbrB [90,94,95,96]. In *Synechocystis* sp. PCC 6803, cyAbrB2 controls the expression of many CCM components that were likewise found to be upregulated in this study (Table 1) [90], while the cyAbrB2 orthologue in *Nostoc* sp. PCC 7120 regulates the expression of FeSOD [96], also upregulated here. *Nostoc* sp. PCC 7120 cyAbrB1 has been recently implicated in transcriptional regulation of heterocyst differentiation [97], demonstrating a role close to the interface of C_i_ and N availability that suggests cyAbrB1/2 were likely active in the transcription regulation observed in the current study.

Given the transcriptional activation of NtcA, the master regulator of N metabolism, by high levels of 2OG upon a shift to low N (high C/N ratio) [7,98,99], it is widely assumed that a decrease in CO_2_ leads to a decline in NtcA activity by decreasing the abundance of the 2OG–NtcA–PipX activator complex [100]. In the current study, lower NtcA activity was indeed evident in the downregulation of *nir* genes encoding NO_3_^−^ uptake and metabolism (Table 1), as well as downregulation of the *nir* co-activator *ntcB* (Table 3; reviewed in [101]). This transcriptional regulation would effectively bring N metabolism into alignment with decreased C metabolism after CO_2_ stepdown, despite the presence of N sources in the BG11 media. Overall, nearly 50% of genes downregulated in the current study have NtcA-binding promoters [71], although many NtcA-regulated genes, such as those involved in the uptake of NH_3_ and urea, regulation of GS-GOGAT enzymes, as well as NtcA itself [71,102,103,104], were not DE after 1 h in low CO_2_. The current work may therefore include only the early NtcA regulon. The NtcA-activated differentiation of vegetative cells to heterocysts occurs over approximately 24 h, via a cascade of transcriptional regulation that includes early upregulation of the co-activator *nrrA* [33]. The rapid downregulation in the current work of some heterocyst regulators, including *nrrA*, may block the commencement of heterocyst differentiation in response to relative N excess over C after CO_2_ stepdown, and may signal an eventual decrease in the small number of heterocysts that are known to occur under high C/N [105]. Downregulation of NtcA activity in the current study appears to indicate a decline in 2OG levels after only 1 h in low CO_2_, although an increased concentration of NH_3_ derived from 2PG metabolism can also explain suppression the NtcA regulon under low CO_2_ (low C/N ratio) [83]. N excess under low CO_2_ is also evident in the −2.2 FC downregulated expression of cyanophycinase chb2 (*all0571*; not shown), which is regulated by NrrA, suggesting a decrease in the mobilisation of stored N under low CO_2_ (reviewed in [106]). It can also be argued that an increase in the ADP/ATP ratio under CO_2_ stepdown, caused by decreased photosynthetic electron transport and rapid changes in metabolism, increases the abundance of both the ADP–PII–PipX complex and the inactive form of NtcA [6].

The strong downregulation of operons involved in ferrous Fe import and Fe–S cluster assembly in the current work (Table 4) suggests a connection between cellular Fe homeostasis and C/N balance in *Nostoc* sp. PCC 7120, which has been explored [107]. The current results indicate a CO_2_ stepdown-induced cellular excess of Fe and other transition metals, which may be due to downregulation of Fe-rich PSI complexes (Table 2) [108] and/or an excess of reductant caused by insufficient electron sinks. Both conditions pose the danger of ROS formation, evidenced by upregulated expression of SOD, peroxiredoxin and protein chaperones.

### 4.2. Altered C/N Balance Modulates the Energetic Strategy of Nostoc sp. PCC 7120

**Figure 1:**
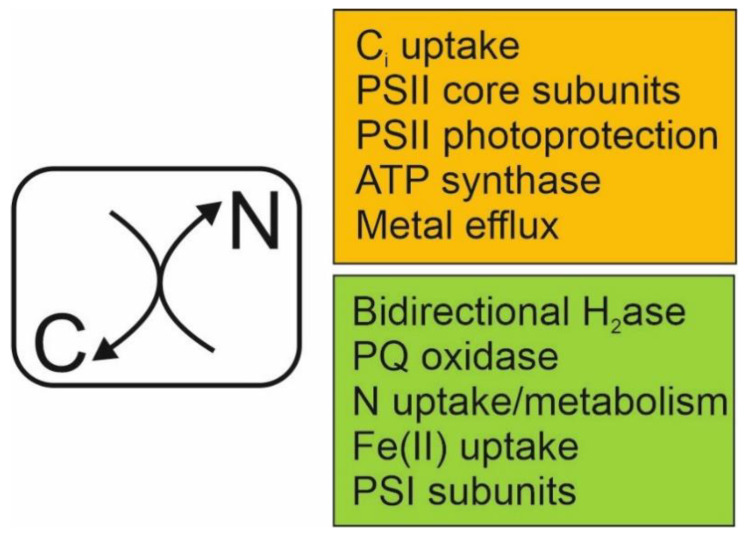
The transcriptional response of *Nostoc* sp. PCC 7120 cells to a change in the cellular concentrations of carbon and nitrogen (C/N balance).Decrease in the external CO_2_ concentration of cultures causes a decline in the cellular C/N balance signalled by an increased production of 2PG and decreased 2OG levels relative to N. This metabolic change leads to upregulation of genes encoding processes depicted in orange, and downregulation of genes encoding processes depicted in green.

**Figure 2:**
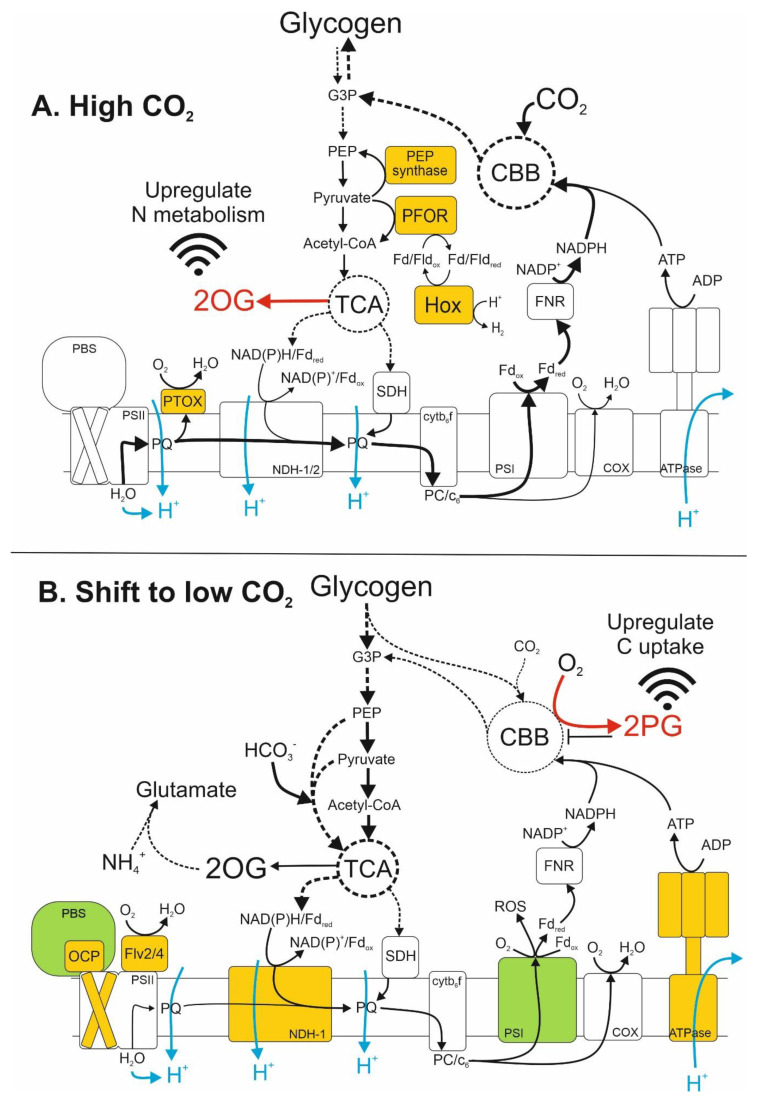
Schematic representation of the interactions between photosynthetic/respiratory electron transport and accumulation of 2OG and 2PG for signalling the C/N balance in *Nostoc* sp. PCC 7120 cells. Scheme is based on global transcriptomic profiling of *Nostoc* sp. PCC 7120 cultures under high CO_2_ conditions and after CO_2_ stepdown. (**A**) Under 3% CO_2_, efficient photosynthetic electron transport and Calvin–Benson–Bassham (CBB) cycle activity enables gluconeogenesis of glyceraldehyde-3-phosphate (G3P), leading to accumulation of carbohydrate storage in the form of glycogen. Glycolysis of glycogen stores and/or photosynthate supplies pyruvate to the incomplete tricarboxylic acid (TCA) cycle, which produces reductant, ATP and succinate to drive respiratory electron transport through NAD(P)H-dehydrogenase (NDH) and succinate dehydrogenase (SDH), as well as other cellular processes. The TCA cycle also generates 2-oxoglutarate (2OG), which accumulates under high CO_2_ due to a relative shortage of NH_4_^+^ and glutamine. 2OG operates as a signal for upregulating genes involved in N uptake and metabolism. Strong expression of phosphoenolpyruvate (PEP) synthase converts pyruvate to PEP. Pyruvate:ferredoxin/flavodoxin oxidoreductase (PFOR) converts pyruvate to acetyl-CoA, reducing oxidised ferredoxin/flavodoxin (Fd/Flv_ox_) that is consumed by bidirectional hydrogenase (Hox) for storage of excess energy as H_2_. Strong expression of plastoquinone terminal oxidase (PTOX) maintains redox homeostasis of the plastoquinone (PQ) pool during high photosynthetic electron transport, while cytochrome c6 oxidase (COX) maintains the redox poise of lumenal electron carriers cytochrome c_6_ (c_6_) and plastocyanin (PC). Genes encoding factors coloured orange are highly expressed under 3% CO_2_ and strongly downregulated by the shift to 0.04% CO_2_. (**B**) After 1 h in 0.04% CO_2_, a deficiency of CO_2_ electron acceptors leads to oxygenation of Rubisco, causing photorespiration that produces 2-phosphoglycolate (2PG). The CBB cycle and other metabolic pathways are inhibited by 2PG, which also signals upregulation of the transcriptomic response to low CO_2_. Low CBB activity causes over-reduction of the photosynthetic electron transport chain, leading to the production of reactive oxygen species (ROS) at photosystem I (PSI) and downregulation of PSI subunits. Increased reducing pressure on photosystem II (PSII) also causes upregulation of the PSII repair cycle and upregulation of PSII photoprotection by flavoproteins (Flv2/4) and orange carotenoid proteins (OCPs), as well as downregulation of phycoerythrocyanin in the phycobilisome (PBS). Glycolysis triggered by decreased CO_2_ assimilation provides substrates for anaplerotic supplementation of the CBB and TCA cycles, including the production of oxaloacetate from PEP and bicarbonate (HCO_3_^−^). TCA cycle activity also produces 2OG for glutamate production from excess ammonium (NH_4_^+^) resulting from the shift from 3% to 0.04% CO_2_. Genes encoding factors coloured orange or green are upregulated or downregulated, respectively, 1 h after the shift from 3% to 0.04% CO_2_. Black arrows indicate the movement of electrons or ATP, dashed arrows summarise multiple enzymatic reactions in carbohydrate metabolism, blue arrows indicate the movement of protons. The red arrow in (**A**) shows the production of 2OG by TCA cycle activity, the red arrow in (**B**) shows the production of 2PG by Rubisco oxygenation in the CBB cycle and the black bar in (**B**) indicates CBB inhibition by 2PG.

The current study shows that a stepdown from 3% CO_2_ in enriched air to 0.04% CO_2_ (atmospheric) for just 1 h led to substantial reprogramming of global gene expression in *Nostoc* sp. PCC 7120 cultures. As discussed above, most of the transcriptional changes observed here can be directly attributed to metabolite signalling instigated by the alteration of the cellular C/N balance (Figure 1), initiated by the decrease in CO_2_ concentration. Furthermore, these results highlight rapid transcriptional reprogramming of photosynthesis and energy metabolism in *Nostoc* sp. PCC 7120 in response to CO_2_ levels (Figure 2). High CO_2_ in light promotes a high rate of photosynthesis and the storage of photosynthate in the form of glycogen [13,109,110,111]. Strong expression of PEP synthase and PFOR under these conditions indicate glycolysis/gluconeogenesis through the metabolism of pyruvate, which is consumed in the TCA cycle to facilitate respiratory electron transport and to provide 2OG for amino acid synthesis (reviewed in [112,113]). Therefore, growth under 3% CO_2_ somewhat resembles photomixotrophy, with photosynthetic and glycolytic metabolisms occurring concomitantly, even though glucose was not externally provided to cultures. Under these conditions, the cells experience a high C/N, which is evident in the relatively high expression of N metabolic genes, reflecting a cellular excess of 2OG [7,98,99]. In high CO_2_, strong expression of Hox, which is important under mixotrophy and N deprivation [55], and PTOX, may provide a system to maintain redox poise [45], and in the case of Hox, also store surplus energy as H_2_ [53]. The transfer of *Synechocystis* sp. PCC 6803 and *Synechococcus elongatus* PCC 7942 cultures from high to low CO_2_ showed that the toxic effects of 2PG transiently block Calvin–Benson–Bassham (CBB) activity [15,20,83] and this is also evident here in the upregulated expression of PSII repair, photoprotection and ROS scavenging enzymes in *Nostoc* sp. PCC 7120, which indicate over-reduction of the photosynthetic electron transport chain. Interestingly, detoxification of 2PG appeared to be downregulated through repression of *alr1004* during CO_2_ stepdown (Table 2), perhaps highlighting the importance of the metabolite for signalling during the early stages of C_i_ deprivation. We also observed downregulation of the terminal proteins in the phycobilisomes, suggesting modified harvesting of light energy to alleviate excitation pressure on the photosynthetic system. Under these conditions, inhibition of photoassimilation is compensated by glycolysis of stored glucose and CBB intermediates, providing an important supply of substrates for anaplerosis of the CBB and TCA cycles during acclimation to the transition [83,114,115]. In the current work, enhanced glycolytic activity is indicated by the upregulation of NDH-1, suggesting an increased reliance on respiratory electron transport for ATP generation, while strong downregulation of PEP synthase and PFOR after CO_2_ stepdown may prevent diversion of pyruvate away from the TCA cycle. Notably, PEP abundance increased substantially in *Synechocystis* sp. PCC 6803 and *Synechococcus elongatus* PCC 7942 after a shift from high to low CO_2_ [20,83], while genes encoding both pyruvate kinase and PFOR were highly expressed in *Synechococcus elongatus* PCC 7942 after long-term acclimation to low CO_2_, but not directly after the transition [20]. These findings support the results of this study and suggest that the metabolism of PEP and pyruvate are tightly regulated after the transition to low CO_2_. This may be linked to the role of PEP and pyruvate as substrates to anaplerotic carbon fixation to produce TCA cycle intermediates oxaloacetate and malate (reviewed in [116]). During the adjustment to a low C/N ratio, TCA cycle activity generates 2OG for amino acid synthesis, utilising excess NH_4_^+^ generated through 2PG detoxification [11,83].

This work has revealed rapid transcriptional reprogramming in *Nostoc* sp. PCC 7120 in response to a decrease in C_i_ availability, namely strong upregulation of CCM components and photoprotection, and downregulation of N uptake and early stages of heterocyst differentiation. Despite the vast increase in HCO_3_^−^ uptake, glycolysis of stored C apparently plays an important role in energy metabolism at low CO_2_, likely due to 2PG-induced inhibition of the CBB cycle. A majority of the transcriptional effects induced by low CO_2_ in *Nostoc* sp. PCC 7120 can be attributed to 2PG modulation of CmpR and a putative NdhR homologue; however, the effects of changing abundance of 2OG and NtcA activity after 1 h in 0.04% CO_2_, as well as the roles of other transcriptional regulators cannot be discounted. Finally, this work highlights the sensitivity of *Nostoc* sp. PCC 7120 to factors that influence the cellular C/N balance and demonstrates the speed at which genetic and metabolic reprogramming can take place, allowing rapid acclimation for surviving and thriving in the fluctuating environment.

## Figures and Tables

**Table 1:** Differentially Expressed (DE) Genes Involved in Carbon and Nitrogen Metabolism.

Name ^1^	Gene ID ^1^	Description	Process	Fold Change ^2^	*p*-Value ^3^
*sbt* operon	*all2133–all2134*	Na^+^-dependent bicarbonate permease, P_II_-like regulatory protein	Bicarbonate import	78.8	<0.001
*cmp* operon	*alr2877–alr2880*	ATP-dependent bicarbonate uptake subunits		36.4	<0.001
*bicA* operon	*all1303–all1304*	Na^+^-dependent bicarbonate permease, Na^+^:H^+^ antiporter		8.7	<0.001
*mrp* operon	*all1837–all1843*	Na^+^:H^+^ antiporter subunits	Na^+^ extrusion, pH regulation	5.4	<0.001
*ndhF3*	*alr4156*	NDH–1MS subunit 5	CO_2_ uptake	2.5	0.002
*ndhD3*	*alr4157*	NDH–1MS subunit 4		2.0	<0.001
*cupA*	*alr4158*	NDH–1MS CO_2_ uptake subunit		5.3	<0.001
*nir* operon	*alr0607–alr0612*	Nitrate/nitrite reductase, ATP-dependent nitrate permease	Nitrate/nitrite import and metabolism	−3.5	0.017
*nirB*	*all0605*	Nitrate-dependent expression of *nir* cluster		−2.9	<0.001
*nifB*	*all1517*	Fe–Mo cofactor biosynthesis subunit	N_2_ fixation, heterocyst development and function	−2.0	0.002
*nifH2*	*alr0874*	Fe–S cluster-binding nitrogenase reductase		−3.2	0.024
*hgd, hgl* clusters	*all5341–alr5359*	Heterocyst glycolipid layer biosynthesis		−2.6	0.004
*dev* operon	*alr3710–alr3712*	ATP-binding subunit, membrane transport subunits		−2.1	0.004
*alr1713*	*alr1713*	Similar to Mo-dependent nitrogenase, C-terminus	Unknown	−5.6	0.002
*asr1714*	*asr1714*	Uncharacterised protein		−5.8	<0.001

^1^ Shaded cells represent operons or clusters of neighbouring genes; ^2^ Fold changes of genes upregulated or downregulated in low CO_2_, compared to high CO_2_, are coloured orange or green, respectively. In cases of multiple genes, average fold changes are shown; ^3^ *p*-values determined by moderated *t*-test. In cases of multiple genes, largest *p*-value is shown.

**Table 2:** Differentially Expressed (DE) Genes Involved in Photosynthesis and Respiration.

Name ^1^	Gene ID ^1^	Description	Process	Fold Change ^2^	*p* Value ^3^
*psbAII*	*alr3727*	Photosystem II D1 protein	PSII electron transport	6.9	<0.001
*psbAIII*	*alr4592*	Photosystem II D1 protein		1.9	0.003
*psbAIV*	*all3572*	Photosystem II D1 protein		3.7	<0.001
*psbD*	*alr4548*	Photosystem II D2 protein		3.4	<0.001
*psaA*	*alr5154*	Photosystem I core protein A1	PSI electron transport	−1.9	0.035
*psaB1*	*alr5155*	Photosystem I core protein A2		−1.9	0.038
*psaB2*	*alr5314*	Photosystem I core protein A2		−2.2	0.031
*psaC*	*asr3463*	Photosystem I Fe-S subunit		−2.2	<0.001
*psaD*	*all0329*	Photosystem I reaction centre subunit 2		−2.7	<0.001
*psaE*	*asr4319*	Photosystem I subunit E		−2.2	0.001
*psaI*	*asl3849*	Photosystem I subunit I		−2.5	0.012
*pasK*	*asr4775*	Photosystem I subunit K		−3.1	0.004
*psaM*	*asr4657*	Photosystem I subunit M		−2.3	0.005
*flv2*	*all4444*	Flavodiiron protein	Other photosynthetic/respiratory electron transport	23.7	<0.001
*all4445*	*all4445*	Unknown protein		31.8	<0.001
*flv4*	*all4446*	Flavodiiron protein		16.7	<0.001
*isiB*	*alr2405*	Flavodoxin		2.9	0.003
*cytA*	*alr4251*	Cytochrome c_6_		−2.2	0.011
*flv1B-flv3B*	*all0177–all0178*	Flavodiiron protein (heterocyst-specific)		−2.0	0.004
*ptox*	*all2096*	Alternative plastoquinone oxidase		−65.0	<0.001
*ftsH*	*alr1261*	FtsH protease	PSII turnover	2.3	<0.001
*ftsH2*	*all3642*	FtsH protease		2.4	<0.001
*pec* operon	*alr0523–alr0527*	Phycoerythrocyanin synthesis	Metabolism/binding of light-harvesting pigments	−3.1	0.015
*chlL, chlN* operon	*all5076–all5078*	Protochlorophyllide reductase, ATP-binding protein		−3.3	<0.001
*chlG*	*all4480*	Chlorophyll synthase 33 kDa subunit		2.2	0.003
*hemH*	*alr4616*	Ferrochelatase		8.6	<0.001
*ocp*	*all3149*	Orange carotenoid-binding protein		23.9	<0.001
*ocp-like*	*all4941*	Orange carotenoid protein-like		3.1	0.010
*asl3726*	*asl3726*	CAB/ELIP/HLIP superfamily		9.8	<0.001
*asr5262*	*asr5262*	CAB/ELIP/HLIP superfamily		8.6	0.005
*atpase* cluster	*all0004–all0010*	ATP synthase subunits	ATP synthesis	2.7	<0.001
*ndh-1* operon	*alr0223–alr0227*	NDH-1 complex subunits	Electron and proton transport	2.3	0.005
*ndh-1* operon	*all3840–all3842*			2.1	0.001
*ndhB*	*all4883*			2.5	0.003
*ndhN*	*alr4216*			1.9	0.001
*alr1004*	*alr1004*	Alanine-glyoxylate transaminase	Glycolate metabolim	−2.7	<0.001
*ndbA*	*all1553*	NDH-2 NAD(P)H:PQ reductase	Respiration	−2.1	<0.001
*hupS*	*all0688*	Uptake hydrogenase, small subunit	H_2_ uptake/evolution	−2.5	0.002
*nifJ/PFOR*	*alr1911*	Pyruvate-ferredoxin/flavodoxin oxidoreductase		−42.4	<0.001
*hox* clusters	*alr0750–all0752* *alr0760–alr0766*	Bidirectional hydrogenase subunits, assembly and regulation		−73.9	0.002
*ppsA*	*all0635*	Phophoenolpyruvate synthase	Glycolysis	−107.4	<0.001

^1^ Shaded cells represent operons or clusters of neighbouring genes; ^2^ Fold changes of genes upregulated or downregulated in low CO_2_, compared to high CO_2_, are coloured orange or green, respectively. In cases of multiple genes, average fold changes are shown; ^3^ *p*-values determined by moderated *t*-test. In cases of multiple genes, largest *p*-value is shown.

**Table 3:** Differentially Expressed (DE) Genes Encoding Transcription Regulators.

Name	Gene ID	Description	Process	Fold Change ^1^	*p* Value ^2^
*cmpR*	*all0862*	LysR-type transcriptional regulator	Regulates *cmp* cluster	3.1	<0.001
*sigB*	*all7615*	Group 2 sigma factor	Response to stress	4.6	<0.001
*sigB3*	*all7608*	Group 2 sigma factor		3.8	<0.001
*C-hik31* operon	*all7583–all7584*	Two-component sensor His kinase, response regulator	Regulation of central metabolism in response to glucose, low O_2_	2.8	<0.001
*P-hik31* operon	*alr1170–alr1171*			1.6	0.001
*all7523*	*all7523*	TetR family regulator	Unknown	2.4	0.046
putative *ndhR* orthologue	*all4986*	LysR-type transcriptional regulator	Repression of CCM expression	−146.2	<0.001
*ntcB*	*all0602*	LysR-type transcriptional regulator	Co-activation of *nir* operon	−2.4	0.002
*devH*	*alr3952*	CRP family transcriptional regulator	Het glycolipid biosynthesis	−2.2	0.006
*patB/cnfR*	*all2512*	Heterocyst patterning	Heterocyst development	−2.4	0.001
*nrrA*	*all4312*	OmpR family regulator		−2.1	<0.001

^1^ Fold changes of genes upregulated or downregulated in low CO_2_, compared to high CO_2_, are coloured orange or green, respectively. In cases of multiple genes, average fold changes are shown; ^2^ *p*-values determined by moderated *t*-test. In cases of multiple genes, largest *p*-value is shown.

**Table 4:** Differentially Expressed (DE) Genes Involved in the Transport and Metabolism of Metals.

Name	Gene ID	Description	Process	Fold Change ^1^	*p* Value ^2^
Fe(II) transport operon	*alr2118–asr2120*	Ferrous iron transporter subunits	Periplasmic iron import	−42.6	<0.001
*suf* operon	*alr2492–alr2496*	ATPase, iron and sulphur transfer	Fe–S cluster assembly, transfer	−3.8	<0.001
Metal efflux cluster	*all7606–all7611*	Proton extrusion, cation efflux	Metal cation efflux, cellular metal homeostasis	3.1	<0.001
Metal efflux cluster	*all7616–all7619*	Cadmium/nickel/zinc/cobalt efflux system		3.9	<0.001
Metal efflux cluster	*all7629–all7633*	Cadmium/nickel/zinc/cobalt efflux system		5.0	<0.001
Cu^2+^ efflux cluster	*alr7634–alr7636*	Putative copper efflux		5.1	0.002

^1^ Fold changes of genes upregulated or downregulated in low CO_2_, compared to high CO_2_, are coloured orange or green, respectively. In cases of multiple genes, average fold changes are shown; ^2^ *p*-values determined by moderated *t*-test. In cases of multiple genes, largest *p*-value is shown.
